# Localization of tamoxifen in human breast cancer tumors by MALDI mass spectrometry imaging

**DOI:** 10.1186/s40169-016-0090-9

**Published:** 2016-03-10

**Authors:** Ákos Végvári, Alexander S. Shavkunov, Thomas E. Fehniger, Dorthe Grabau, Emma Niméus, György Marko-Varga

**Affiliations:** Department of Biomedical Engineering, Clinical Protein Science and Imaging, Lund University, BMC D13, 221 84 Lund, Sweden; Department of Pharmacology and Toxicology, University of Texas Medical Branch, Galveston, TX USA; Department of Oncology and Pathology, Clinical Science, Lund University, Lund, Sweden; Department of Surgery, Skåne University Hospital, Lund, Sweden

**Keywords:** Human breast cancer, Tamoxifen treatment, MALDI-mass spectrometry imaging, Estrogen receptor stratification, Drug localization, Drug quantification

## Abstract

**Background:**

Tamoxifen is used in endocrine treatment of breast cancer to inhibit estrogen signaling. A set of stratified ER-positive and ER-negative tumor sections was subjected to manual deposition of tamoxifen solution in order to investigate its spatial distribution upon exposure to interaction within thin tissue sections.

**Methods:**

The localization of tamoxifen in tumor sections was assessed by matrix assisted laser deposition/ionization mass spectrometry imaging. The images of extracted ion maps were analyzed for comparison of signal intensity distributions.

**Results:**

The precursor ion of tamoxifen (*m/z* 372.233) displayed heterogeneous signal intensity distributions in histological compartments of tumor tissue sections. The levels of tamoxifen in tumor cells compared with stroma were higher in ER-positive tissues, whereas ER-negative tissue sections showed lower signal intensities in tumor cells.

**Conclusions:**

The experimental model was successfully applied on frozen tumor samples allowing for differentiation between ER groups based on distribution of tamoxifen.

**Electronic supplementary material:**

The online version of this article (doi:10.1186/s40169-016-0090-9) contains supplementary material, which is available to authorized users.

## Background

Breast cancer is the most common malignancy among women in the Western world, affecting approximately every tenth woman. In the past decades, there has been considerable improvement in the prognosis, which enhanced survival, although still 1500 women die each year from the disease in Sweden only. Breast cancer is treated locally with surgery and radiotherapy while systemic treatment includes chemotherapy, endocrine therapy and targeted drugs. The endocrine treatment involves blocking the estrogen receptor (ER) pathway and is a targeted treatment inhibiting the binding of estrogen to the ER (using tamoxifen) or by removing the ligand estrogen (using aromatase inhibitors or oophorectomy). Despite new promising drugs, patients are still recurring due to a failure to respond to treatment. Drug resistance is one possible cause to therapy failure, however, the mechanisms behind drug resistance are not fully understood. Additionally, with the exception for ER and HER2 status, there are no predictive factors used in the clinical routine for guidance in treatment sensitivity.

The proteins involved in drug uptake in breast cancer tissue have shown to play an active role in drug resistance [1], and we have in a previous study discovered glycoproteins involved in response to tamoxifen, distinguishing recurring from non-recurring breast cancer patients [2]. Altered expression profiles of target proteins have been studied using radioligand binding to characterize receptor distribution and functional state [3]. Treatment failure may be explained by different patterns of drug uptake distribution. Localization of administered drugs is mostly studied in animal models, which may provide limited insights to their molecular mechanism in humans. With the currently available biobanking samples we applied a recently developed methodology to investigate whether ER stratified human tumors can be distinguished upon exposure to tamoxifen in solution.

Matrix-assisted laser desorption/ionization-mass spectrometry imaging (MALDI-MSI) is an established technology used to localize molecules of various nature in tissue sections in order to investigate their spatial and temporal distributions in organs or whole body [4–7]. The technology of MALDI-MSI was introduced in 1994 [8] and localizations of protein in brain sections was demonstrated soon after [9]. Since then MALDI-MSI has been applied to lateral and temporal localization of other molecules, including lipids [10], and drug compounds [11]. Due to the broad availability of MALDI mass spectrometers and the relatively simple sample preparation required, the methodology has been intensively developed in drug distribution analyses [11]. The detection of an orally administered drug compound directly in mouse tumor tissue surface using MALDI-MS was demonstrated by acquiring data in an array of positions [12]. Quantitative analyses of drug molecules using MSI technology was investigated and compared with standard LC–MS/MS methods [13–17]. MALDI-MSI has been typically applied for drug imaging of low-molecular-weight compounds administered to experimental animals, utilizing the exceptional advantage that no chemical derivatization is required [18]. High resolution MSI significantly improved the localization of drug metabolites [19–22]. The approach allows simultaneous detection of pharmacologically active compounds and their metabolites [4, 19], supporting pharmacokinetic (PK) and pharmacodynamic (PD) developments in the pharmaceutical industry, e.g., contributing to PK screening [23, 24]. Recently, we have introduced a novel methodology based on experimental models, when tissue sections from untreated animals were deposited with or submerged in drug solutions prior to MALDI-MS imaging analysis [25, 26]. This method has revealed specific localizations of compounds in heterogeneous tumor sections.

In this study, we investigated the localization of tamoxifen in five ER-positive and five ER-negative breast tumor sections using an experimental model by MALDI-MSI. In this model, tamoxifen was manually overlaid and incubated on the tissue surface, after which the localization and quantification of its precursor ion was determined.

## Methods

### Patients

Frozen human breast cancer tissue was provided by the South Swedish Breast Cancer Tumor Bank. Ten samples with freshly frozen tumor tissues (ca. 5 × 5 × 5 mm each) from breast cancer patients were selected and ER was evaluated using immunohistochemistry (IHC) by a pathologist at the Department of Oncology and Pathology in Lund. The definition of ER-positive is >10 % positive cells and negative is ≤10 % positive cells. We chose to include ER-positive tumors with >75 % positive cells and ER-negative with 0 %. Five of the tumors were ER-positive and five were negative. One ER-positive and one ER-negative tumor were excluded due to poor tissue quality with few cancer cells. The ethical permission for this study was approved by the Ethical Committee of Lund (LU-240-01).

### Sample preparation

Tamoxifen (CAS 10540-29-1; Mw = 371.515 g/mol; IUPAC name: (*Z*)-2-[4-(1,2-diphenylbut-1-enyl)phenoxy]-*N*,*N*-dimethylethanamine) was obtained in form of pills (Zitazonium™), each containing 10 mg active compound among Mg-stearate, A type carboxymethyl-Na starch, povidone K25, microcrystalline cellulose, potato starch and lactose-monohydrate (108.2 mg). One pill was crushed and 1 mL DMSO was added in order to dissolve the powder. The suspension was then further diluted with distilled water in several steps until obtaining a clear solution at 0.1 mg/mL.

To characterize ionization properties, a dilution series of tamoxifen (0.001–10 µg/mL) was applied on a MALDI target plate and dried droplet sample spots were prepared by adding 1 µL of 3.5 mg/mL α-cyano-4-hydroxycinnamic acid (CHCA, from Sigma-Aldrich, St. Louis MO) matrix solution in 50 % acetonitrile (AcN)/0.1 % trifluoroacetic acid (TFA) to 1 µL tamoxifen solution.

Human breast tumor samples were cut at 15 µm thickness at −20 °C using a cryostat (Leica CM1950, Leica Biosystems, Nussloch, Germany) after transferring them from −80 °C and collected on glass slides (25 × 75 × 1 mm Superfrost Ultra Plus, Thermo Scientific). For MALDI-MS imaging analysis each collected tissue section was treated with 100 % methanol (MeOH) for 5 min and covered with 500 µL of 2 µg/mL tamoxifen solution (aq.) followed by incubation in a humidity chamber for 1 h at room temperature. After removal of the excess solution, the sections were extensively washed with distilled water and then dried in air. The CHCA matrix solution at 7.5 mg/mL concentration (in 50 % AcN/0.1 % TFA) was then sprayed stepwise onto the tissue surface using an airbrush (Aztek A470, Testor Corp., Rockford IL).

### MALDI mass spectrometry imaging analysis

The MALDI-MS imaging acquisition was performed using a MALDI LTQ Orbitrap XL mass spectrometer (Thermo Fisher Scientific, Bremen, Germany) with a method that employed 10 laser shots at 10 µJ. Automatic gain control was switched off. The tissue surface was sampled in meandering mode at 40, 50 or 80-µm raster step size. Full scans in profile mode were generated in a mass range from *m/z* 150 to 500 in the Orbitrap at resolution of 60,000 (at *m/z* 400) in positive polarity.

Following data acquisition, the raw files were opened in ImageQuest™ software (Thermo Fisher Scientific, San José, CA) and the precursor mass of tamoxifen (*m/z* 372.233) was extracted showing its localization within the tissue sections. Screen shots were taken of the distribution of tamoxifen precursor ion normalized on total ion count (TIC). For determination of signal response, the precursor ion intensities of tamoxifen were normalized to the CHCA signal (*m/z* 372.092 of [2 M + H]^+^) and plotted against the calculated concentrations (0.001–10 µg/mL), see Additional file 1: Figure S1.

After removal of the matrix, the slides were stained with Mayer’s hematoxylin-eosin (HE). Cover slipped H and E-stained slides were loaded into the slide scanner (Mirax Midi Slide Scanner, Zeiss, Germany) to take detailed images for selection of regions of interest at high resolution [26]. The raw image files were opened in Aperio ImageScope Viewer v12.1 (Leica Biosystems Imaging Inc., Vista, CA), where a photo of the detailed HE scan could be taken and saved as a *.tiff* image file. The same HE images were annotated by a pathologist highlighting tumor cell dense regions using the Path XL web-based software tool. These annotations were transferred and overlaid on to extracted ion maps of tamoxifen for comparison of tumor areas with stroma within each section using the open source image processing software Fiji (ImageJ v2.0.0; http://imagej.net). Outlines of the areas representing the malignant tissue were directly derived from the highlights made by the pathologist. Outlines of the areas representing unaffected stroma were obtained from the images of HE stained tissue, in which the ROIs representing the tumor areas were filled with background color. The images were then converted to binary representation, and “Create Selection” function was applied to outline the tissue sections and create regions of interest which excluded tumor areas and spaces not occupied by cells. The mean intensity values were determined and used for statistical evaluation calculating the *p* value in two-tailed paired *t* test.

## Results

### Characterization of tamoxifen by MALDI-MS

**Fig. 1:**
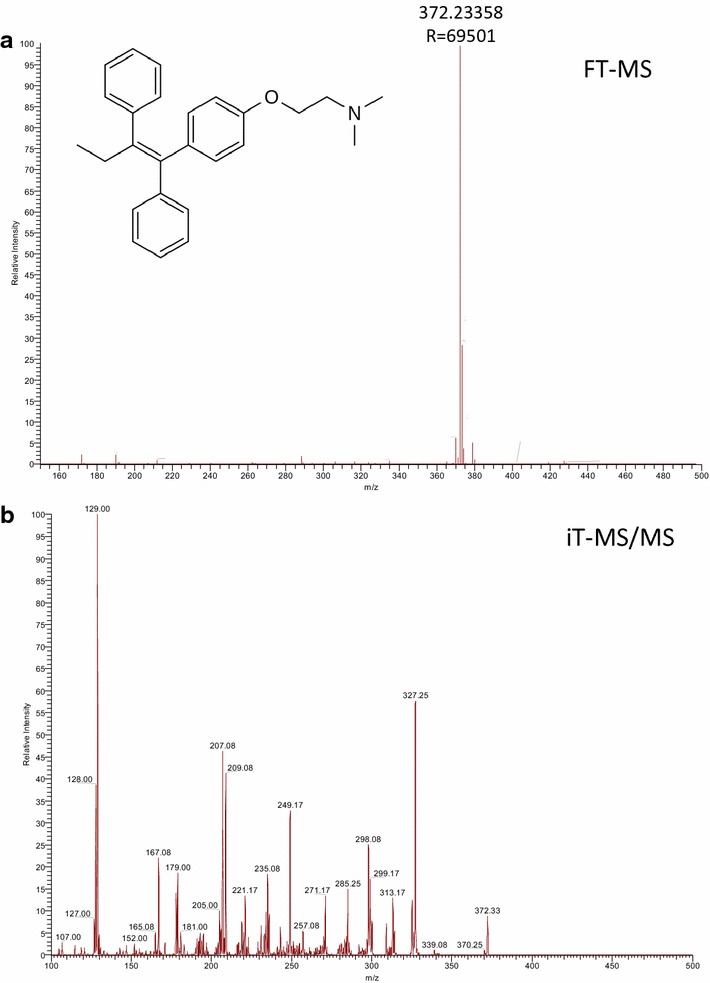
Ionization characteristics of tamoxifen (0.1 mg/mL in water) as measured with 3.5 mg/mL CHCA on a stainless steel MALDI target plate. **a** A full mass spectrum of tamoxifen obtained at 60,000 resolution using the Orbitrap mass analyzer and **b** a tandem mass spectrum of tamoxifen isolating the *m/z* 372.23 and CID fragmented in the linear ion trap mass analyzer

Tamoxifen was obtained in the form of a commercial medicine (Zitazonium™) formulated as tablets with 10 mg active compound. Following initial dissolving of a pill, the entire content was used for determining the ionization properties of tamoxifen on a MALDI LTQ Orbitrap XL mass spectrometer. A singly, positively charged precursor ion of tamoxifen (*m/z* 372.233) was readily observed in full mass spectra as shown in Fig. 1a. Following mass isolation of this peak, the CID fragmentation in the linear ion trap, applying 35 % normalized collision energy, has produced a complex tandem spectrum indicating multiple fragment ions of tamoxifen (see Fig. 1b).

The same precursor ion of tamoxifen (*m/z* 372.233) was observed in tissue sections when a solution of the drug was deposited manually and matrix was sprayed on the surface, as shown in Additional file 2: Figure S2. However, the typical peaks of CHCA were also clearly detectable in these spectra, which was due to the high concentration of matrix required for sufficient ionization on tissue surface. Under such conditions, the well-known ion suppression effect may contribute to the generally lower signal intensities of drug analytes detected in MALDI-MSI experiments. Utilizing the superior mass resolution and accuracy of the Orbitrap mass analyzer, full scan mode was chosen for data acquisition in imaging mode rather than tandem mass spectra.

The signal intensities of tamoxifen precursor ion were measured on a stainless steel MALDI target plate in the concentration range between 0.001–10 µg/mL. It was found that 10 µg/mL tamoxifen could overfill up the Orbitrap (asking for 10^6^ target ions) but good linearity was obtained between 10^0^–10^4^ ng/mL concentration (see Additional file 1: Figure S1). Accordingly, the LOD of tamoxifen was estimated to be less than 1 ng/mL (2.7 nM) on target plate. Due to the different ionization properties of tamoxifen on a tissue surface, this reference correlation was not used to estimate the actual amount of drug in tissue sections. Instead, the selected regions of interests (ROIs) of tumor cell dense and stroma areas were compared based on careful annotations of these regions by a trained pathologist (see “Localization of tamoxifen in human breast tumor sections” section).

### Localization of tamoxifen in human breast tumor sections

**Fig. 2:**
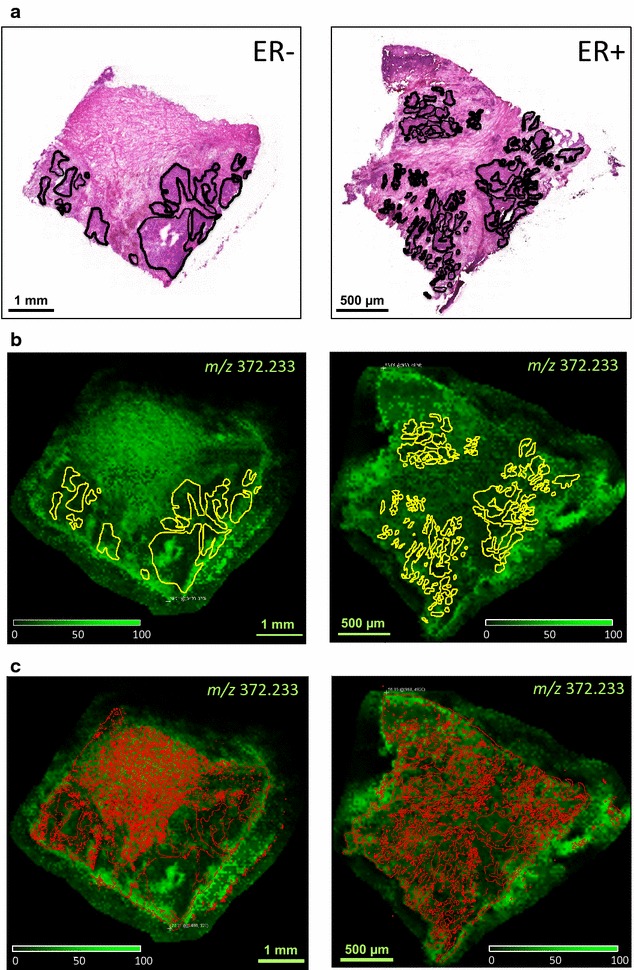
Examples of tamoxifen localization in experimental model analysis showing the precursor ion (*m/z* 372.233) distribution in an ER-negative (*left panels*) and an ER-positive (*right panels*) phenotype tissue section. The *dotted lines* define the areas covered by tumor cells as determined by a pathologist in H and E images (**a**). The extracted ion maps of the tamoxifen precursor ion were normalized on TIC and the same intensity scale was used to compare tumor sections. The tumor and stroma ROIs, that were used for quantitative comparison, were highlighted in *yellow* (**b**) and *red* (**c**), respectively

A visual comparison of tumors suggested that ER-negative tissues had a tendency of larger continuous areas covered by cancer cells in contrast to the ER-positive tumor sections that typically displayed discontinuous, scattered cancer cell patterns (Fig. 2a). Dried breast tumor sections on glass slides were treated with a tamoxifen solution (2 µg/mL) according to the experimental model introduced previously [25, 26]. The general concept of this model is based on manual deposition of a drug on tissue surface and incubation at room temperature before matrix application. Following the sample preparation steps, MALDI-MSI analysis revealed the localization of tamoxifen within tumor sections, showing heterogeneous distributions of its precursor ion that followed histological structures, as typical examples shown in Fig. 2b. Additionally, Additional file 3: Figure S3 presents all breast tumor sections analyzed by MALDI-MSI using identical experimental parameters. In order to compensate for the ion suppression usually associated with MALDI-MS analysis, the precursor ion (*m/z* 372.233) intensities were always normalized on TIC.

In accordance with previous observations [25, 27], in several instances, lower signal intensities of tamoxifen were observed in tumor cell dense regions as annotated in yellow lines by a trained pathologist (see Fig. 2a). However, the complex patterns of tumor cells, especially in the ER-positive tissue sections, limited the visual comparison of drug signal distributions. We have utilized the thorough pathological annotations and created masks in an imaging software, dividing the area of each tissue section to tumor and stroma cell covered regions. These histology derived ROIs were then transferred onto extracted ion maps of tamoxifen (normalized on TIC) in monochromic MS images. The mean signal intensities in tumor and stromal regions were determined in each tissue section and presented in Table 1. The ratios of the mean signal intensities in these regions were evaluated for differentiating ER-negatives and ER-positives, as data indicated higher signal intensities in tumor cells in three out of four ER-positive samples. The statistical analysis, applying paired *t* test, indicated a significant difference between the ER groups in the investigated samples (p = 0.01). Although, the distributions of tamoxifen signal intensities displayed different profiles, they were always similar in tumor cells and stroma ROIs of the same tissue (see histograms in Additional file 4 Figure S4).

**Table 1:** Summary of image analysis

Sample	Area	Mean signal intensity	Ratio of mean intensities
21296/ER−			
Tumor	20,633	141.956	0.961
Stroma	51,585	147.683	
21498/ER−			
Tumor	46,556	14.861	0.555
Stroma	174,838	26.79	
21505/ER−			
Tumor	57,135	35.279	1.082
Stroma	121,614	32.601	
21524/ER−			
Tumor	41,870	71.572	0.779
Stroma	99,257	91.917	
21506/ER+			
Tumor	4198	60.243	1.138
Stroma	176,973	52.923	
21508/ER+			
Tumor	29,101	62.652	0.849
Stroma	87,341	73.769	
21509/ER+			
Tumor	45,132	14.877	1.278
Stroma	120,119	11.637	
21557/ER+			
Tumor	28,673	28.369	1.151
Stroma	62,461	24.643	

The mean signal intensity of the tamoxifen precursor ion (*m/z* 372.233) was determined in annotated tumor and stroma regions within each tissue section using the normalized MS images. The ratios of mean intensities (tumor/stroma) were calculated for statistical *t* test that showed significant differences between the ER groups (p = 0.01)

## Discussion

We have analyzed ten human breast tumors stratified for ER status, following interaction with a manually deposited tamoxifen solution, as described previously [25, 26]. The application of a homogeneous solution on the tissue surface permitted a free movement of drug molecules, driven by physical transport (diffusion), and allowed for various molecular interactions. Although, the cells in the tissue sections were dead but most of them were cut open by sectioning and became rehydrated during sample preparation, rendering a suitable environment for interactions between drug and protein molecules. We can speculate that proteins with preserved conformation might even display weak biological activity facilitating specific interactions with ligands.

Following incubation in a humidity chamber with controlled conditions to treat all sections identically, including the removal of drug solution and rinsing, the samples were dried in air at room temperature. Similar control was pertained during matrix application, producing homogeneous crystallization with even thickness that was achieved by deposition of the same amount of CHCA over an area in each section. Visual investigation of the matrix layers using a stereo microscope at 5× magnification, could not reveal any profound differences in crystal size or coverage. We have perfected airbrush deposition and achieved good reproducibility in several studies previously and could occasionally recognize tissue structure under the matrix layer in large sections with greater histological heterogeneity only. Ionization efficiency therefore assumed to be similar throughout the experiments, which was seen in TIC images. Furthermore, the tissue sections were fixated to the glass surface using MeOH wash that could effectively remove abundant lipids, normalizing their levels between stroma and cancer cells. Consequently, apparent variations in signals of tamoxifen observed in tumor and stromal cells assumed to be accounted for differential interactions with the ligands, resulting in their accumulation in spaces with higher density of target molecules, such as ER.

In general, it is not known that ER-negative tumors have a more solid way of growing. Although ER-negative tumors are more frequently low differentiated tumors and histological grade 3 indicating a worse prognosis. ER-negative tumors are more frequently proliferating, with a higher Ki67 [18]. Hence, tumors with low differentiation, high histological grade and high proliferations markers may have a tendency of growing more solidly. This is in line with our findings with a tendency of larger cancer cell fields as the pattern of growing in the ER-negative tumors.

The identified precursor mass of tamoxifen (*m/z* 372.233) was extracted and mapped over the tissue sections after MALDI-MSI data acquisition. It was found that the drug signal distributed heterogeneously over the tissue sections showing characteristically higher intensities from stroma compared to tumor cell dense areas typically in ER-negative sections, whereas ER-positive tissue sections displayed an opposite distribution of tamoxifen signals (Table 1). The results of ER-negative tissues agreed well with previous findings when a drug solution was spotted on heterogeneous tumor surface un equal amount at each position [27]. It was hypothesized that tumor cells might had different surface properties regulated by their altered molecular composition as compared with normal (stromal) cells. Additionally, this observation is in agreement with MALDI-MSI data from in vivo animal models displaying drug signals significantly lower in cancer cells [22]. Furthermore, it has been previously shown that the protein expression of ER-positive and ER-negative tumors differed significantly [28]. However, the direct comparison of ER stratified tissues likely reflects the differences in levels of ER expression in tumor cells.

MSI provides a more universal approach to study drug binding to characterize receptor distribution in tissue sections than the radio-ligand method [3], without a need to use radioactive compounds. In case the target receptor is expressed but its function is altered due to mutations [29], the drug binding/retention capacity may be compared for tumor and stroma cells in ER-negative and ER-positive cancer. In this way, the MSI based methodology may serve as a predictor for therapy response revealing with physiological drug distribution that can be studied in patient biopsies following administration. This finding has to be further investigated and verified in future studies with breast cancer samples.

## Conclusions

This proof-of-principle study using sections of stratified breast cancer tumors with manually overlaid tamoxifen has revealed histology dictated spatial localizations of the drug molecules as detected by MALDI-MSI. We have shown distributions of tamoxifen in both ER-positive and ER-negative tumors, detecting the drug at significantly lower intensities in tumor cells compared with stroma in ER-negative samples. However, ER-negative tissue sections displayed larger continuous areas covered with tumor cells than that of ER-positive sections, these later ones displayed accumulated tamoxifen in tumor cells. Based on our findings, this experimental model can be useful in characterizing drugs in tumors and applied to further investigate molecular mechanisms of drug uptake and distribution.

## Additional files

10.1186/s40169-016-0090-9 Quantification of tamoxifen on MALDI target plate using normalization of signal intensity on the [2 M + H]^+^ matrix peak (*m/z* 379.092). A dilution series of tamoxifen solutions was deposited and mixed with the matrix solution to obtain homogenous crystallization before MALDI-MS analysis.
10.1186/s40169-016-0090-9 Ionization characteristics of tamoxifen (0.1 mg/mL in water) as measured with 7.5 mg/mL CHCA directly on a section of human breast tumor. MALDI-MS analysis conditions were identical with those used for MALDI-MSI.
10.1186/s40169-016-0090-9 Localization of tamoxifen in ER-negative (n = 4) and ER-positive (n = 4) breast tumor sections by MALDI-MSI analysis highlighting areas with high cancer cell densities as determined and shown in H and E stained sections. MALDI-MSI data acquisition parameters were identical with those results presented in Fig. 2.
10.1186/s40169-016-0090-9 Distribution of signal intensities of tamoxifen in tumor and stroma ROIs. The mean intensities, as determined by image analysis, are presented in Table 1.
