# Supplementary material for: Localization of tamoxifen in human breast cancer tumors by MALDI mass spectrometry imaging
Source: Clin Transl Med. 2016 Mar 10;5:10. doi: 10.1186/s40169-016-0090-9 (PMC4786513; doi:10.1186/s40169-016-0090-9)
Supplement: Supplementary file 1 — 10.1186/s40169-016-0090-9 Quantification of tamoxifen on MALDI target plate using normalization of signal intensity on the [2 M + H]+ matrix peak (m/z 379.092). A dilution series of tamoxifen solutions was deposited and mixed with the matrix solution to obtain homogenous crystallization before MALDI-MS analysis. [file 40169_2016_90_MOESM1_ESM.pptx]

## Slide 1
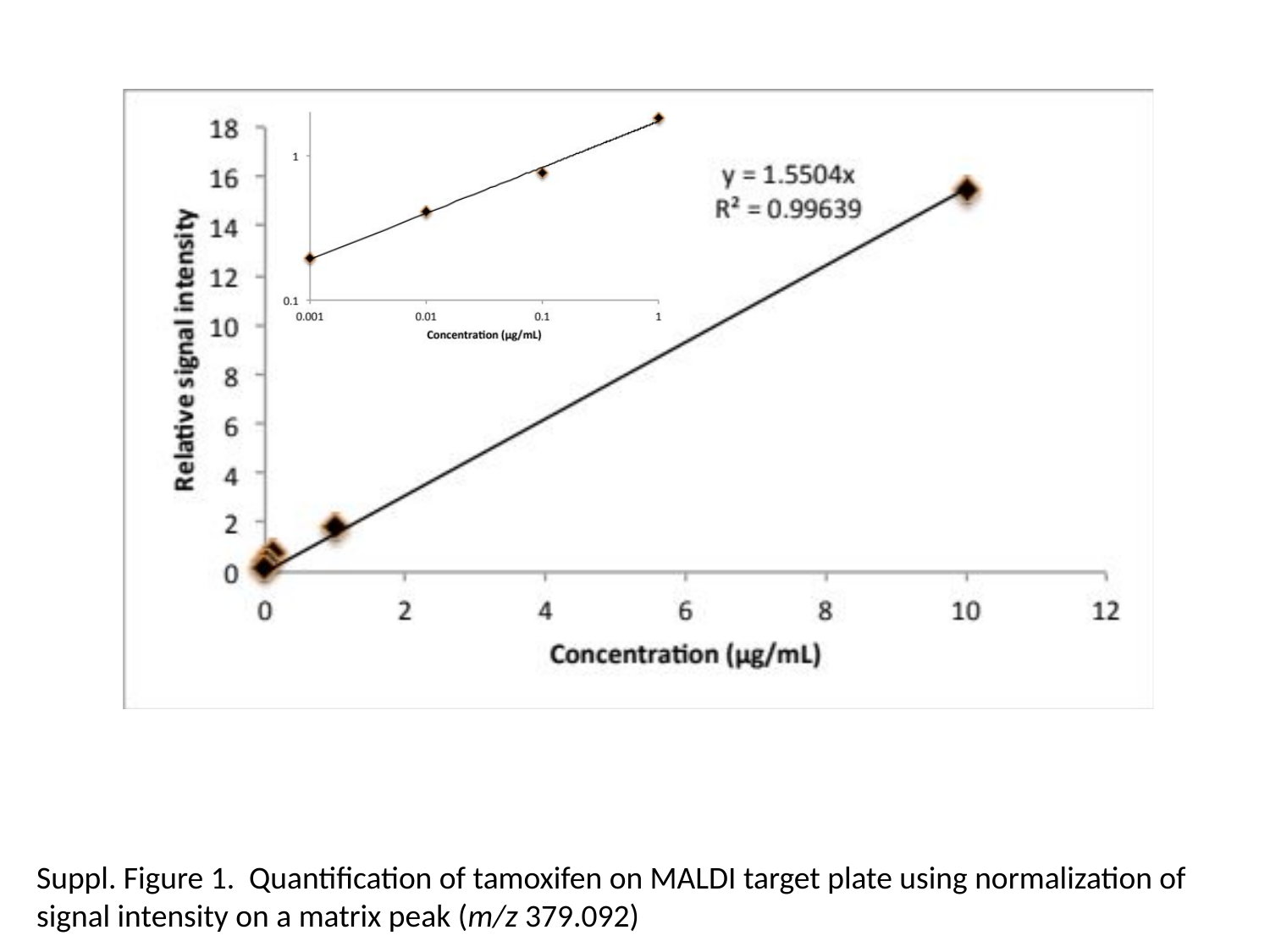

Suppl. Figure 1. Quantification of tamoxifen on MALDI target plate using normalization of signal intensity on a matrix peak (m/z 379.092)
