# Supplementary figures and images for: Localization of tamoxifen in human breast cancer tumors by MALDI mass spectrometry imaging
Source: Clin Transl Med. 2016 Mar 10;5:10. doi: 10.1186/s40169-016-0090-9 (PMC4786513; doi:10.1186/s40169-016-0090-9)

## Slide 1
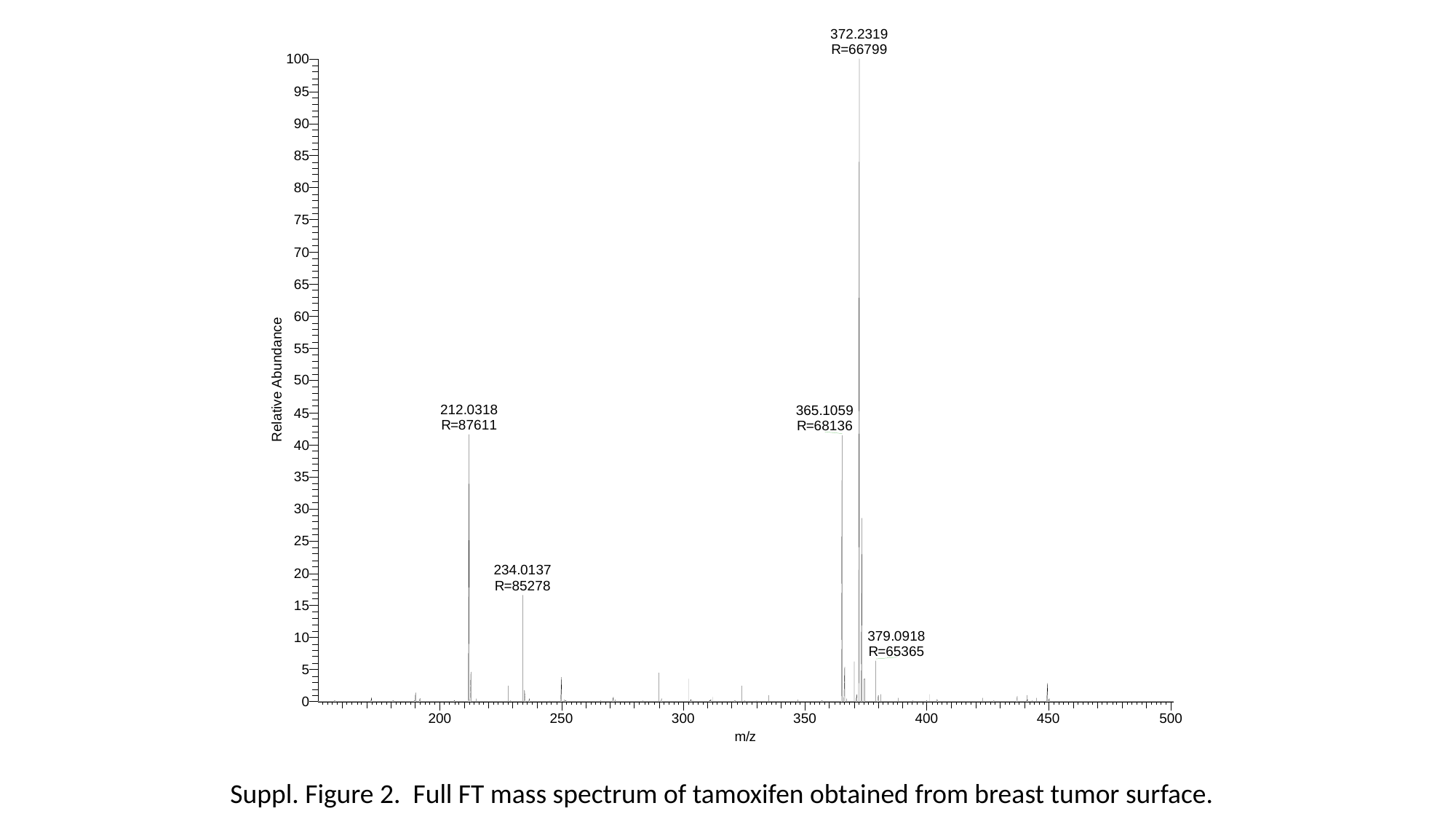

Suppl. Figure 2. Full FT mass spectrum of tamoxifen obtained from breast tumor surface.

Supplement: Supplementary file 2 — 10.1186/s40169-016-0090-9 Ionization characteristics of tamoxifen (0.1 mg/mL in water) as measured with 7.5 mg/mL CHCA directly on a section of human breast tumor. MALDI-MS analysis conditions were identical with those used for MALDI-MSI. [file 40169_2016_90_MOESM2_ESM.pptx]

m/z 372.233/TIC

21498/5 ER-

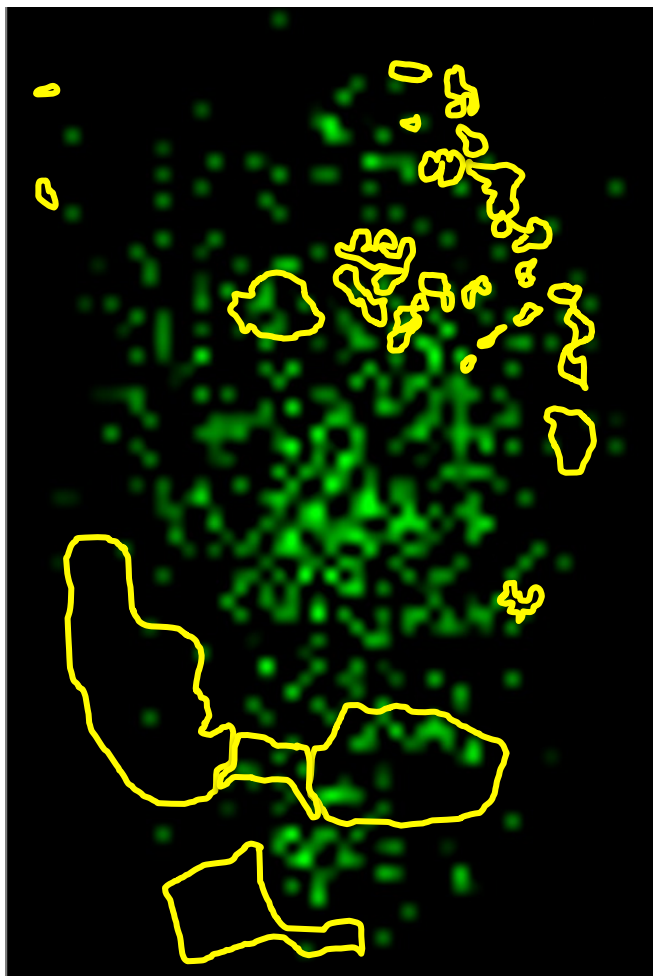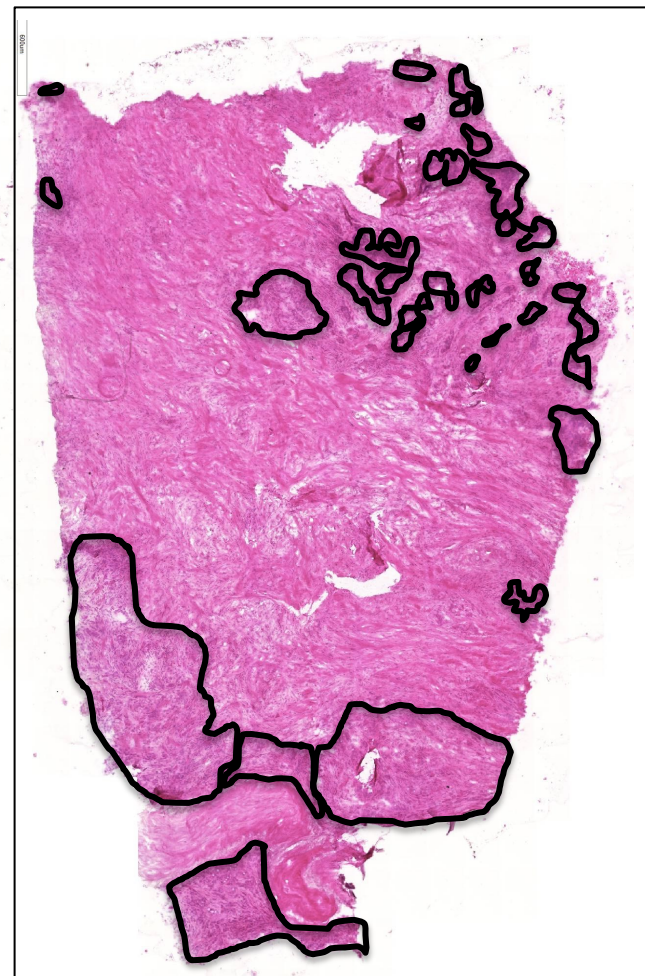

m/z 372.233/TIC

21505/5 ER-

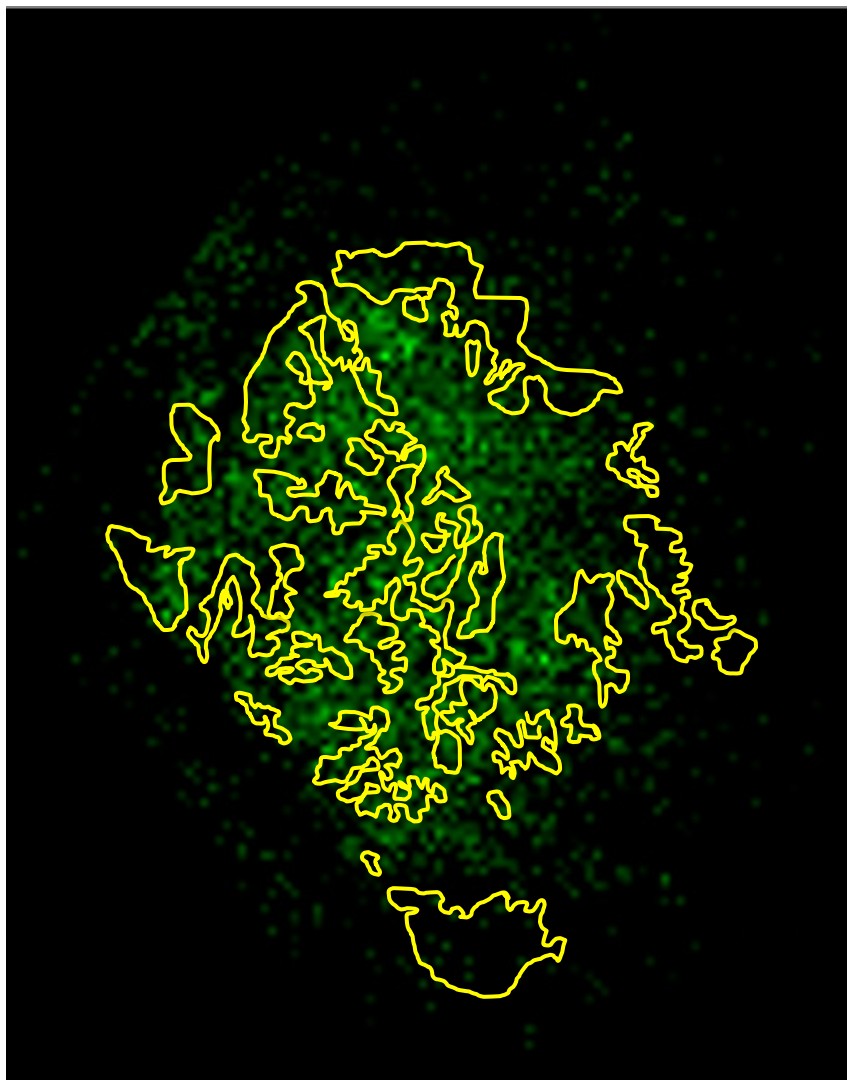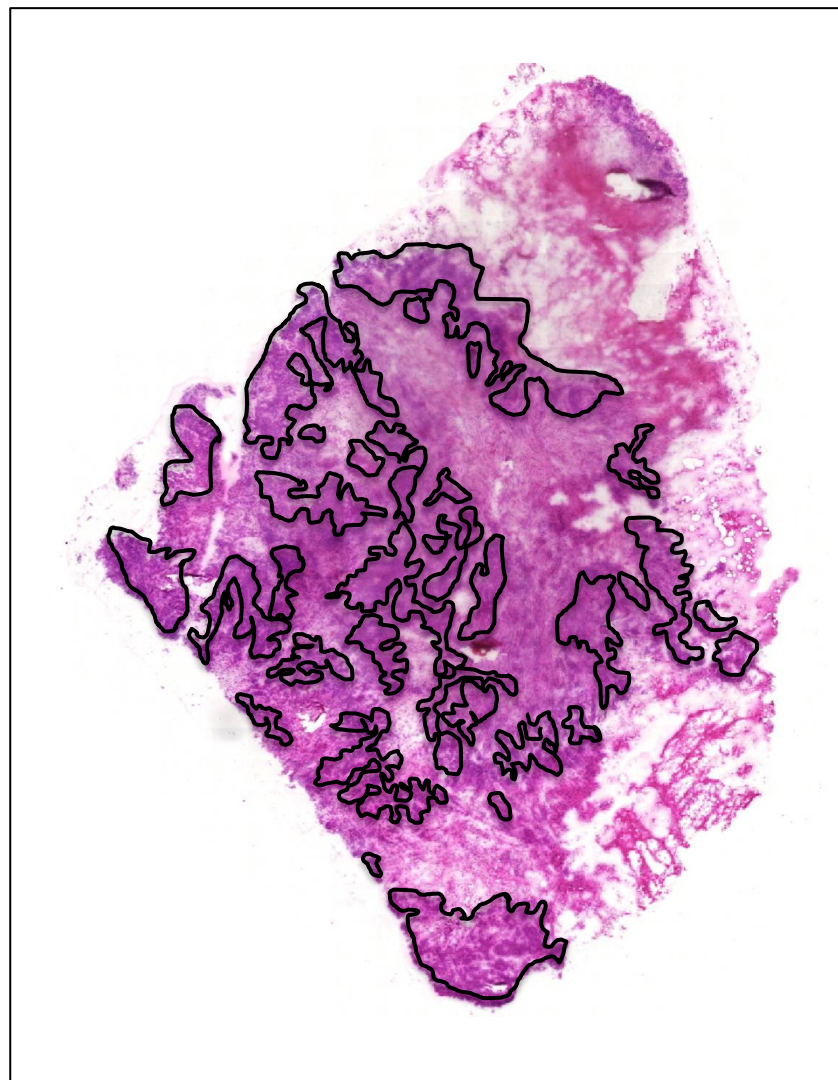

21506/5 ER+

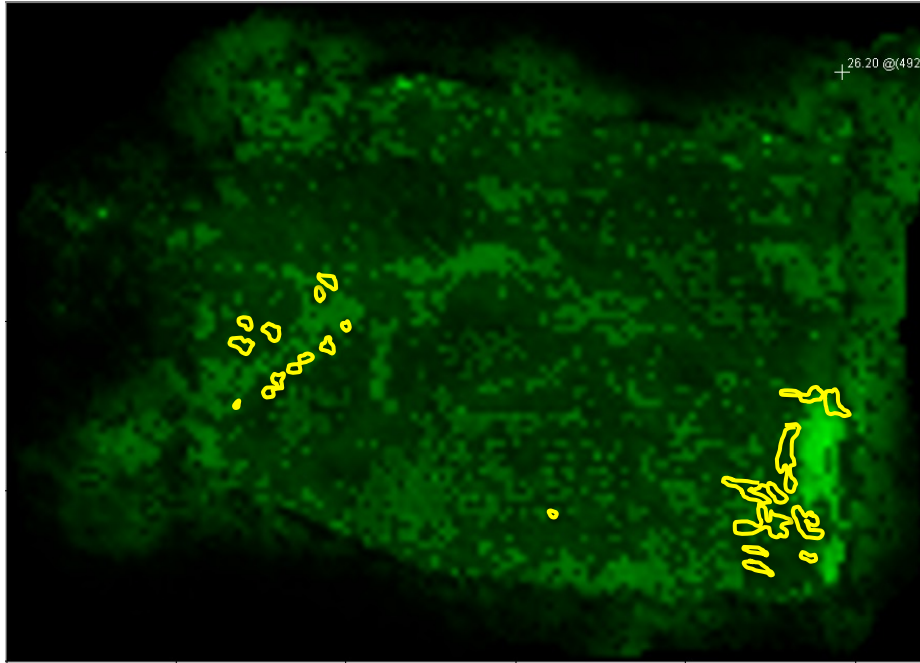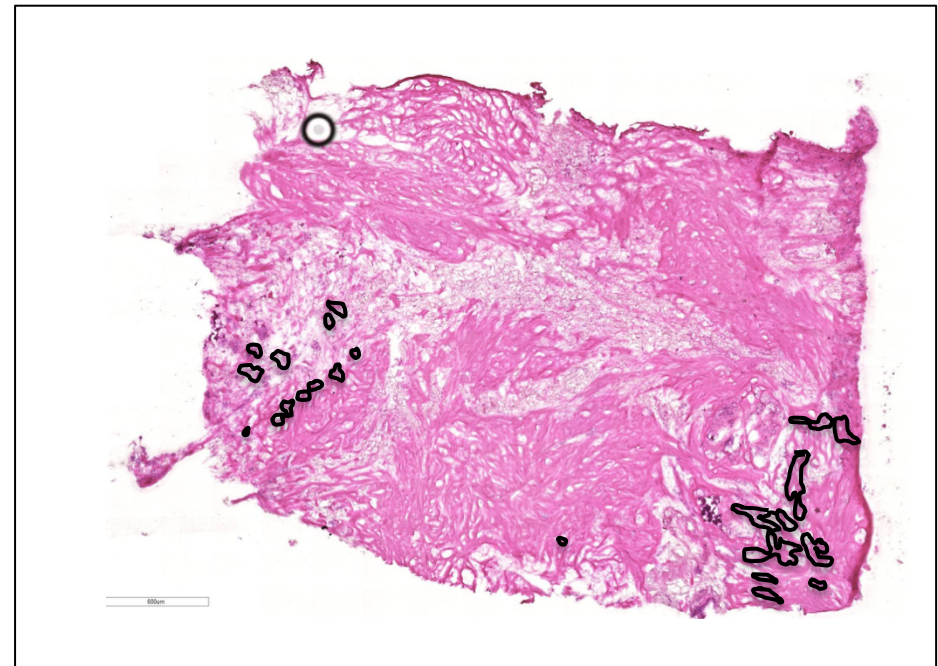

21508/4 ER+

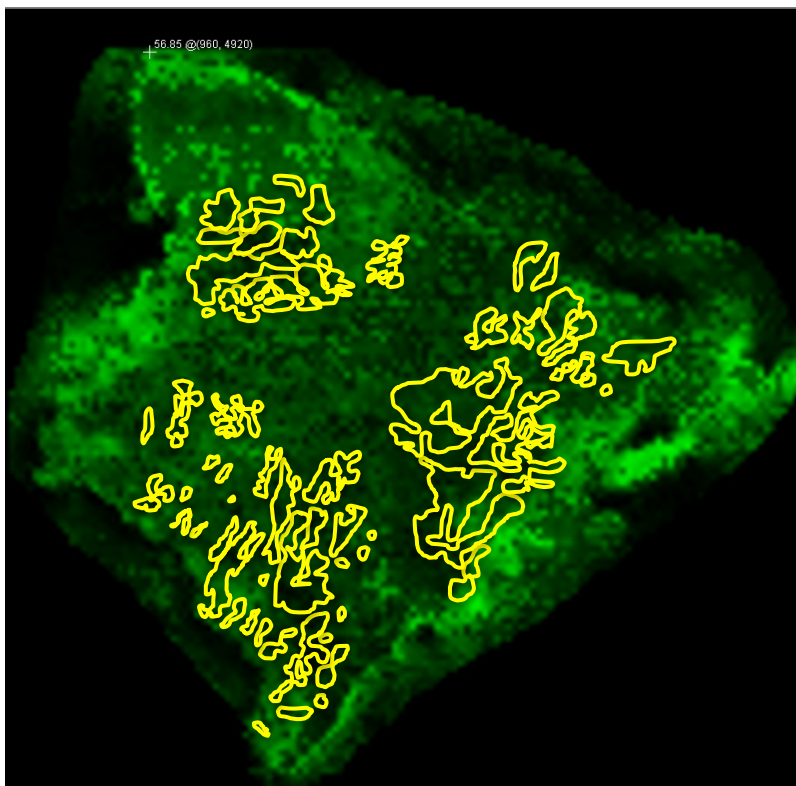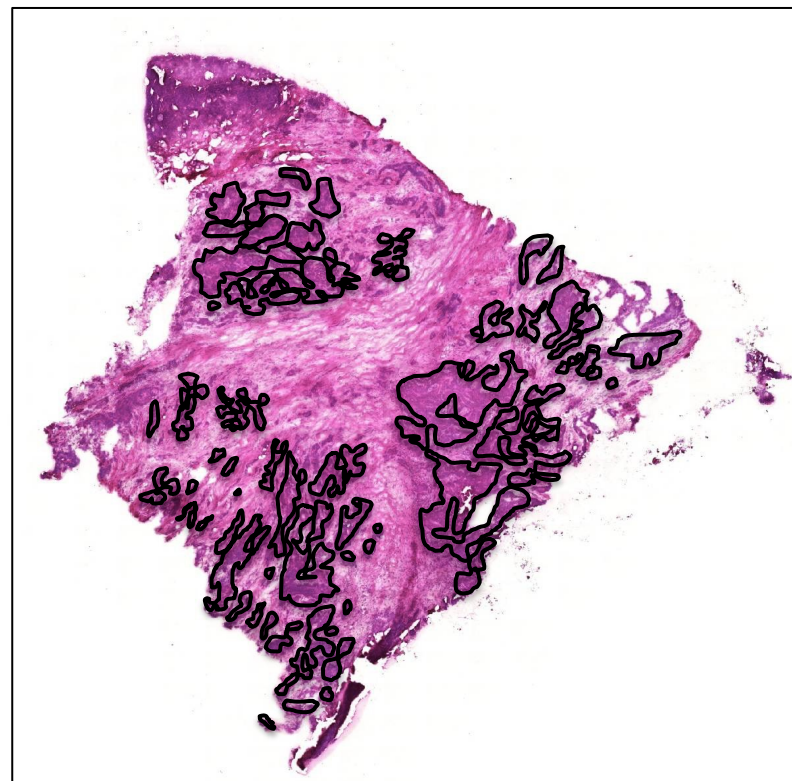

m/z 372.233/TIC

21509/3 ER+

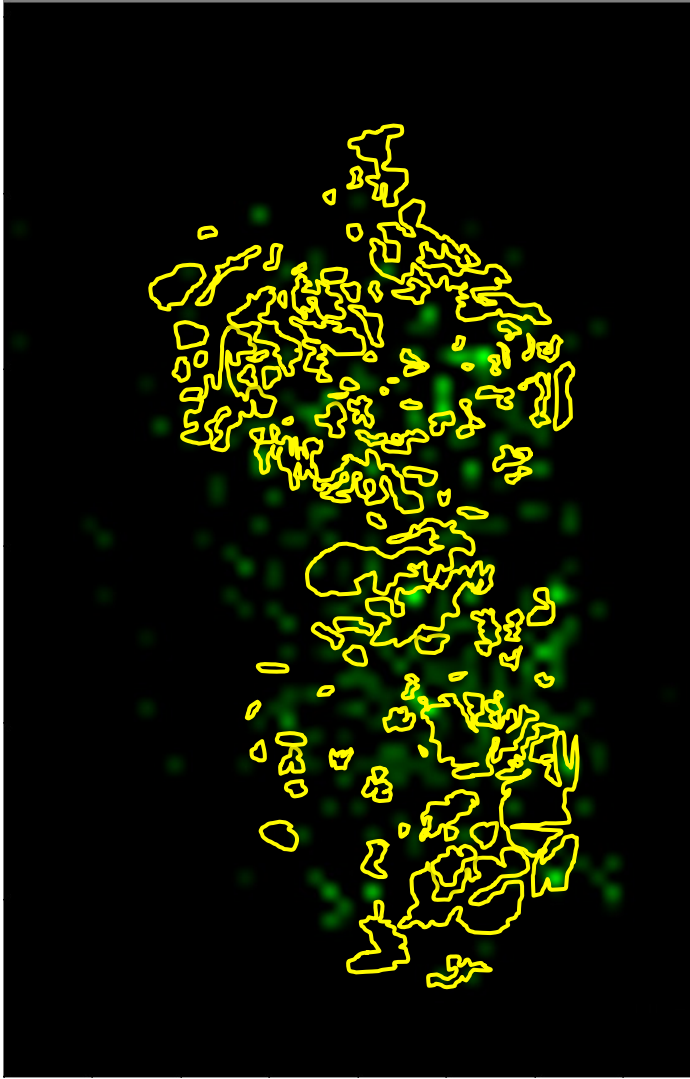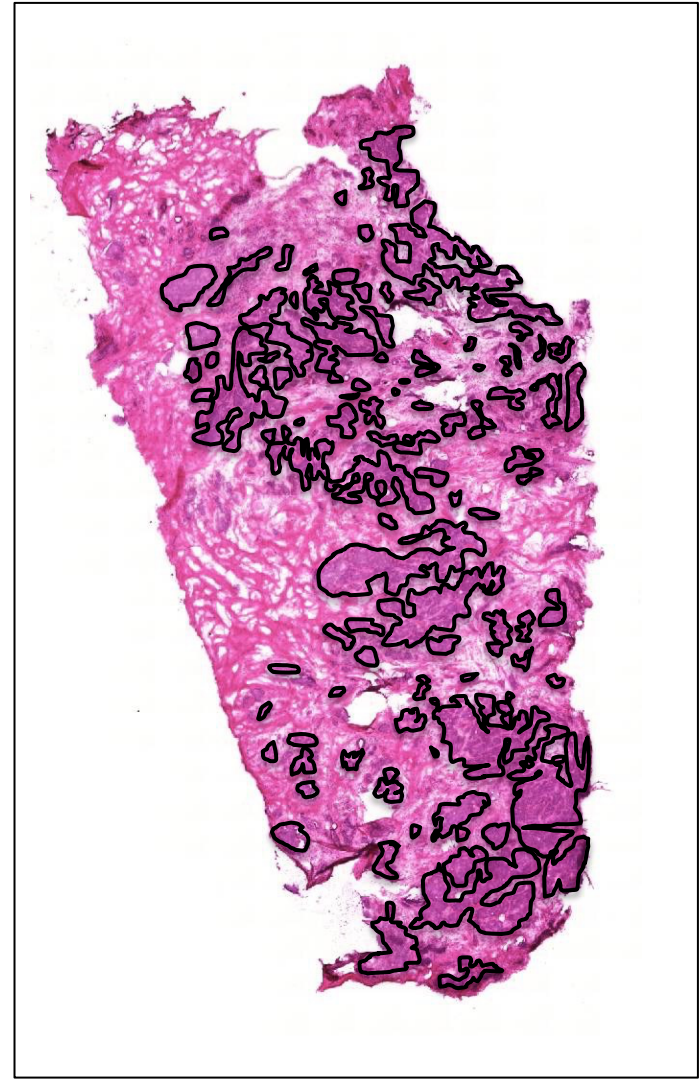

24524/7 ER-

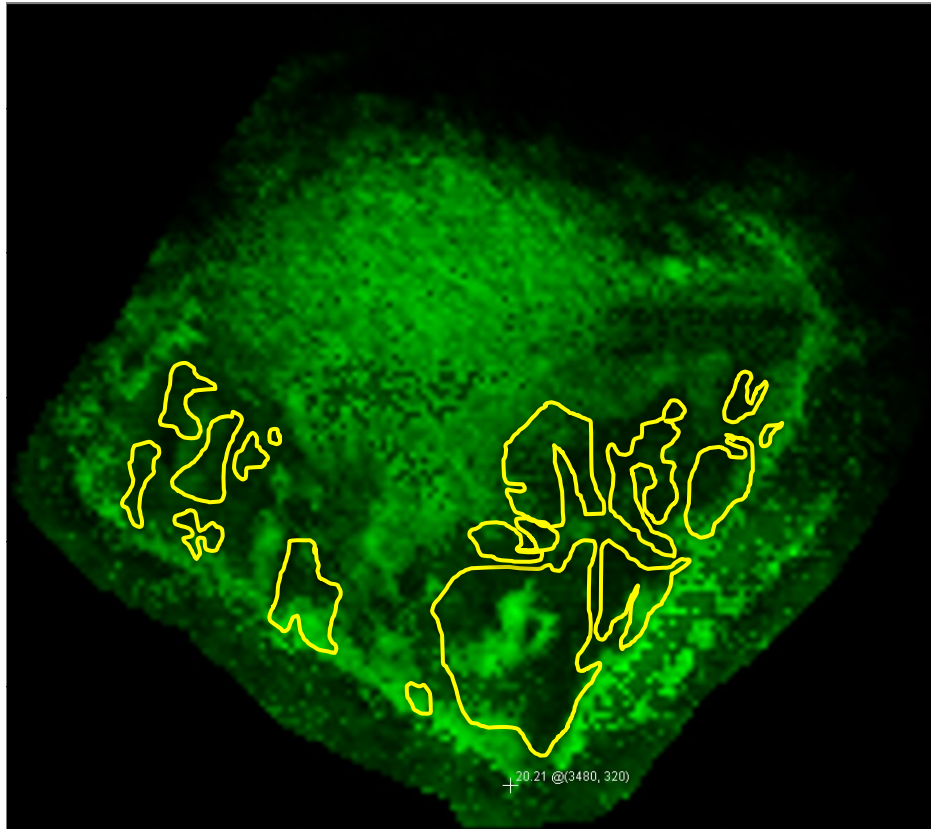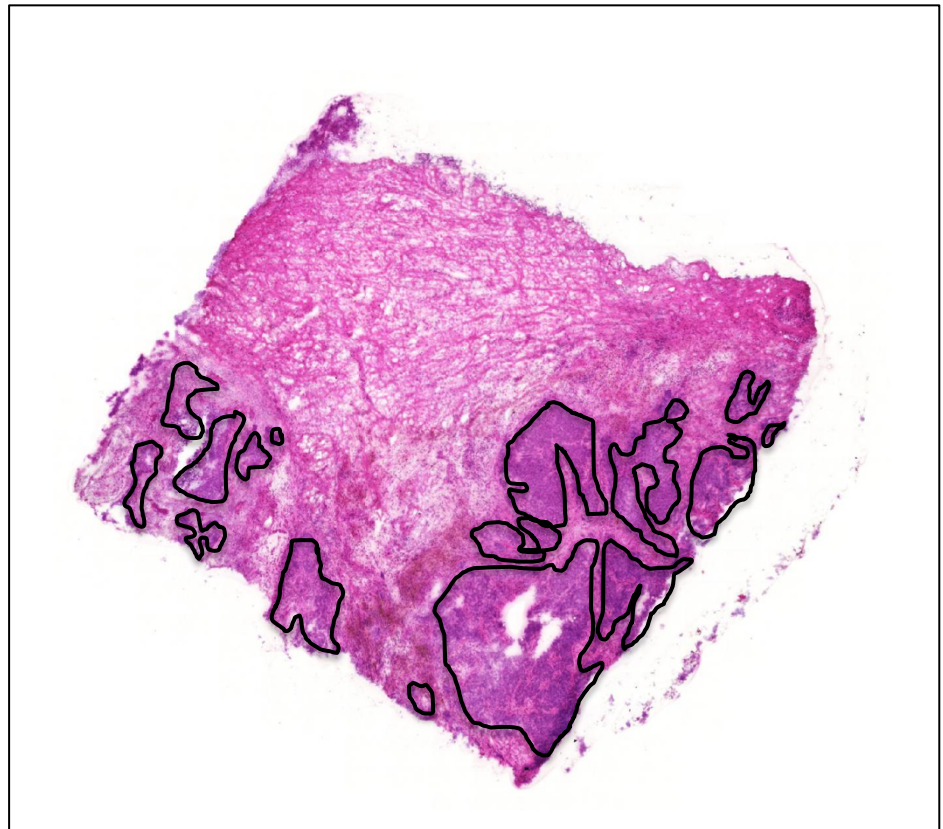

m/z 372.233/TIC

21557/3 ER+

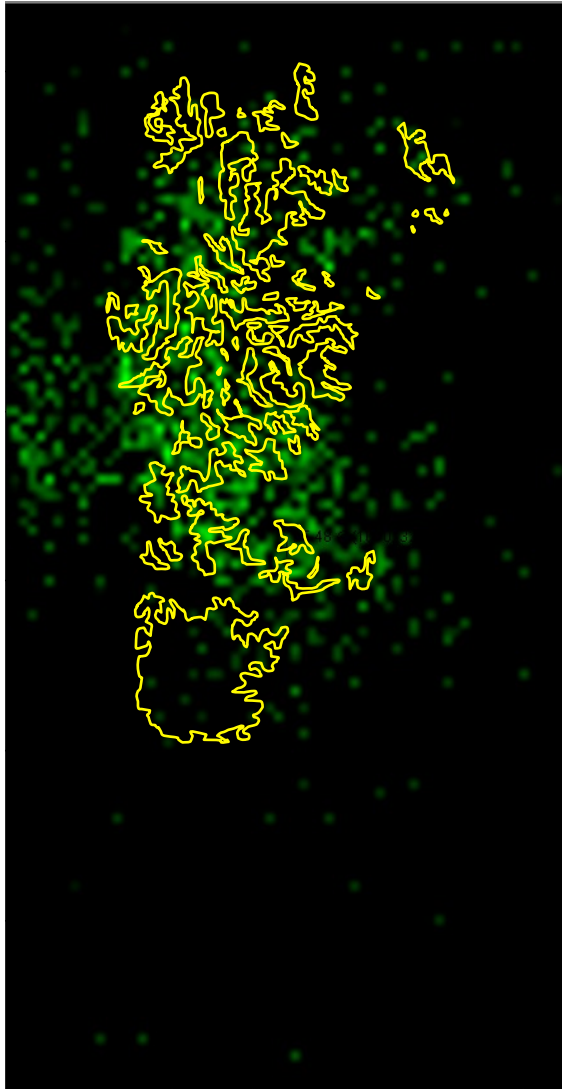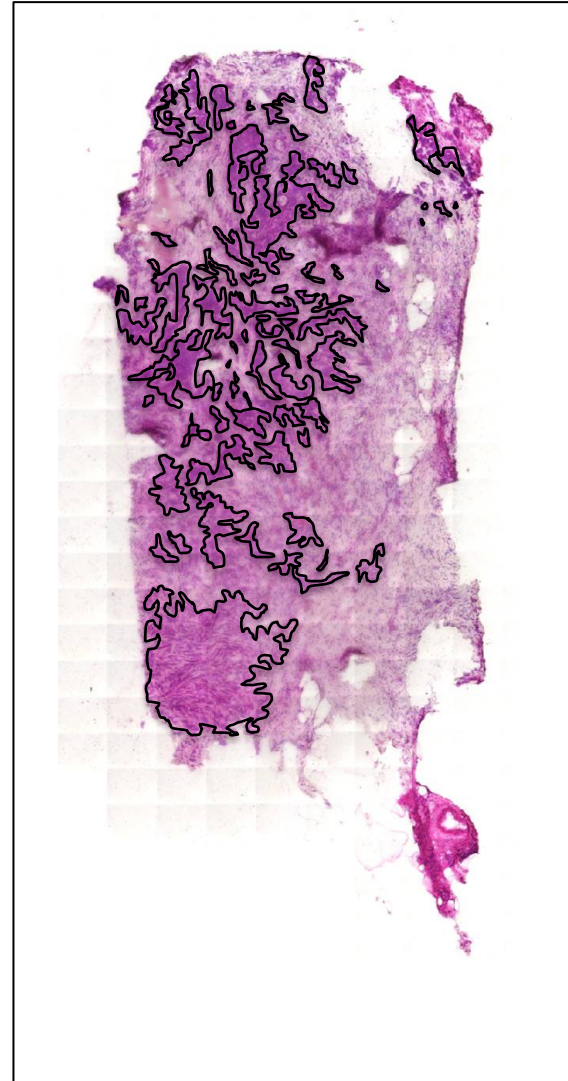

21296/5 ER-

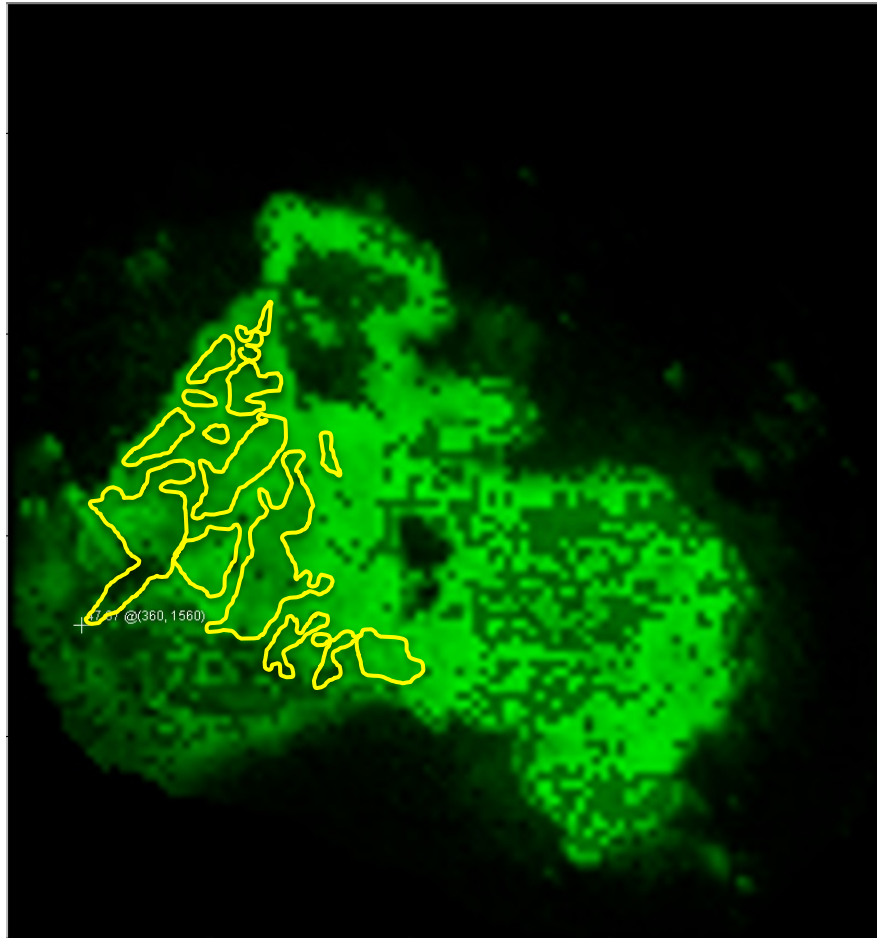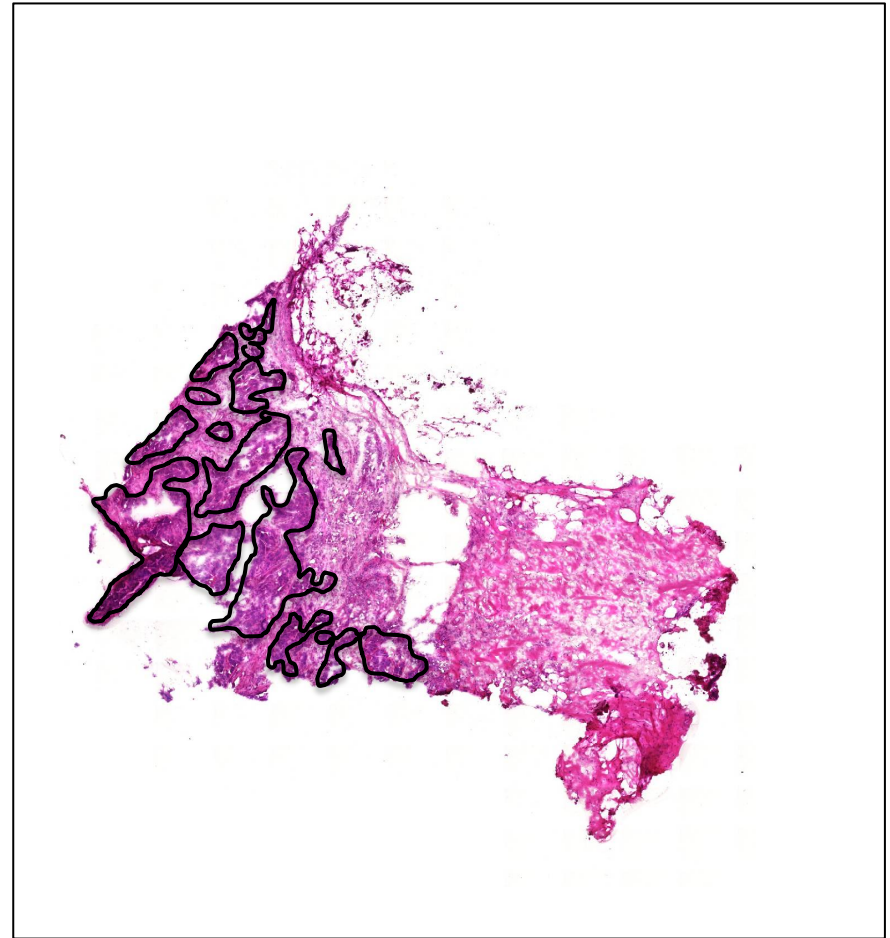

Supplement: Supplementary file 3 — 10.1186/s40169-016-0090-9 Localization of tamoxifen in ER-negative (n = 4) and ER-positive (n = 4) breast tumor sections by MALDI-MSI analysis highlighting areas with high cancer cell densities as determined and shown in H and E stained sections. MALDI-MSI data acquisition parameters were identical with those results presented in Fig. 2. [file 40169_2016_90_MOESM3_ESM.pdf]
