# Supplementary material for: Localization of tamoxifen in human breast cancer tumors by MALDI mass spectrometry imaging
Source: Clin Transl Med. 2016 Mar 10;5:10. doi: 10.1186/s40169-016-0090-9 (PMC4786513; doi:10.1186/s40169-016-0090-9)

21296/-

Tumor

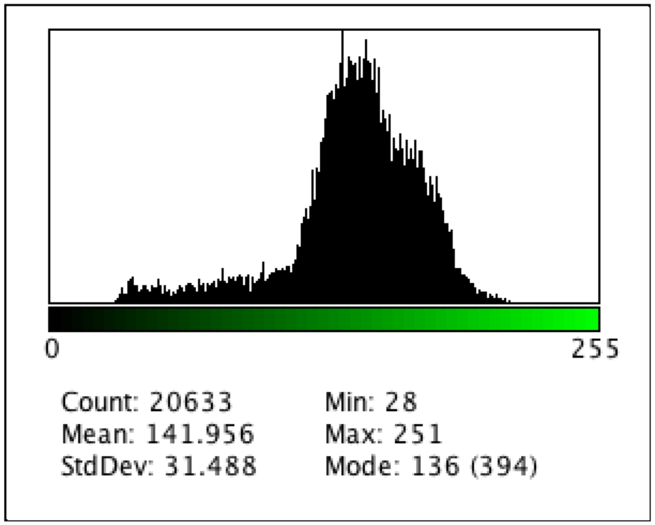

Stroma

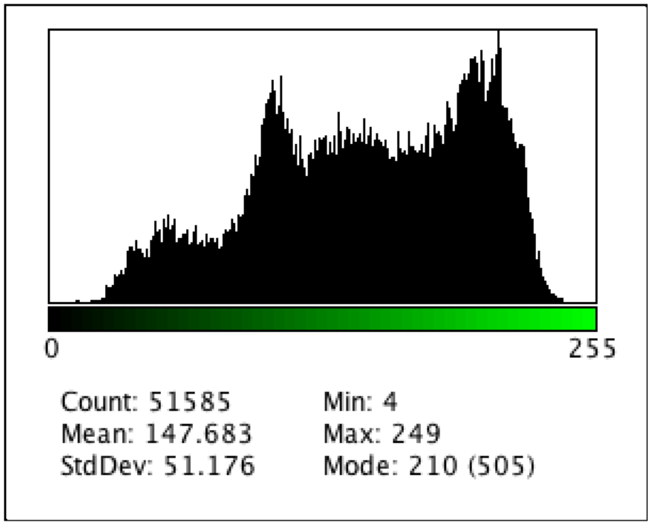

21498/-

Tumor

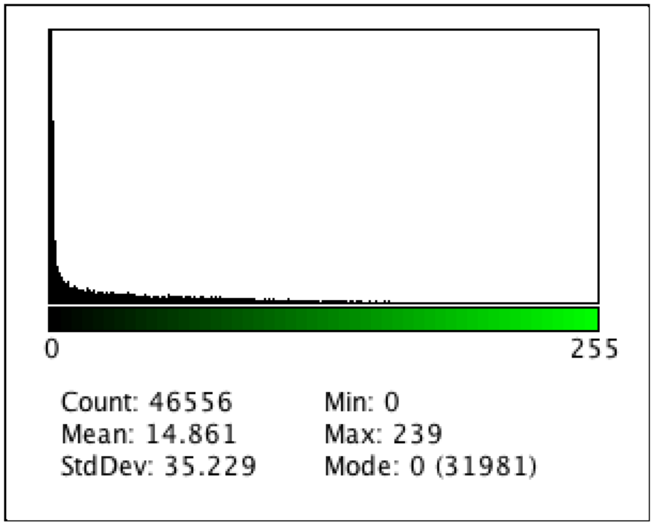

Stroma

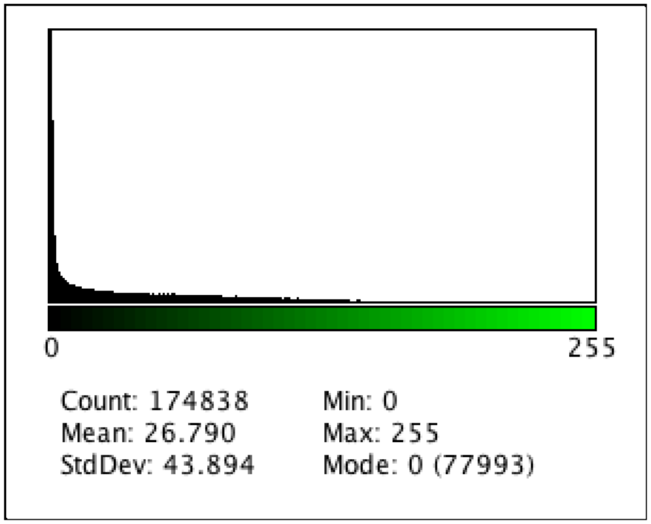

21505/-

Tumor

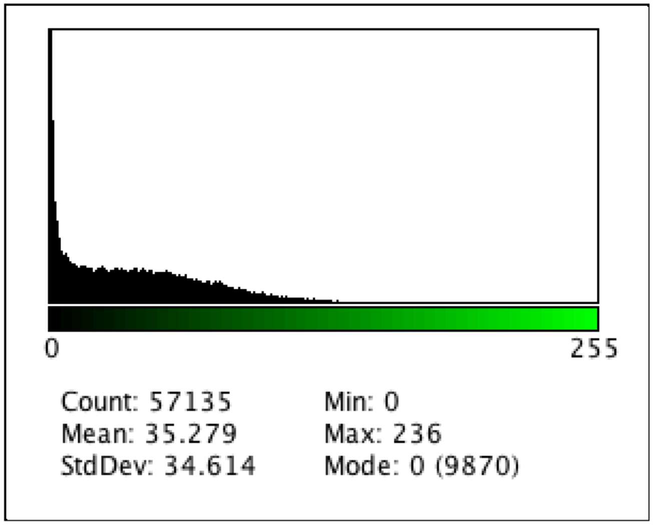

Stroma

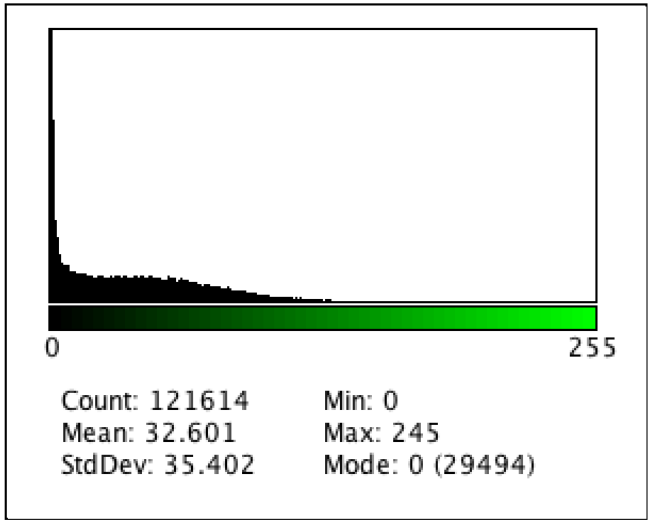

21524/-

Tumor

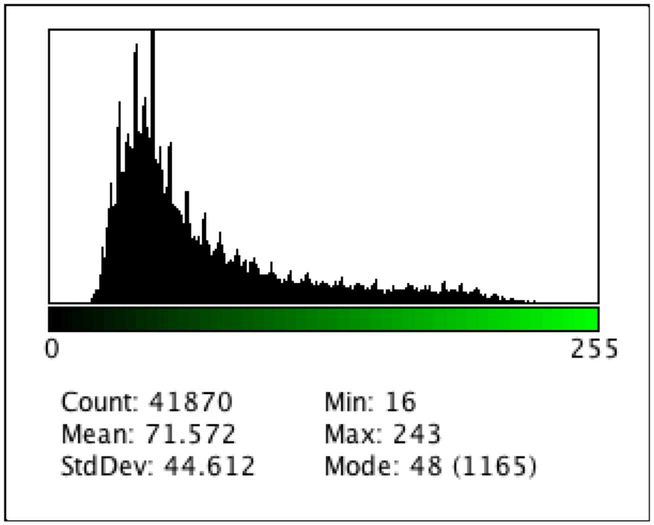

Stroma

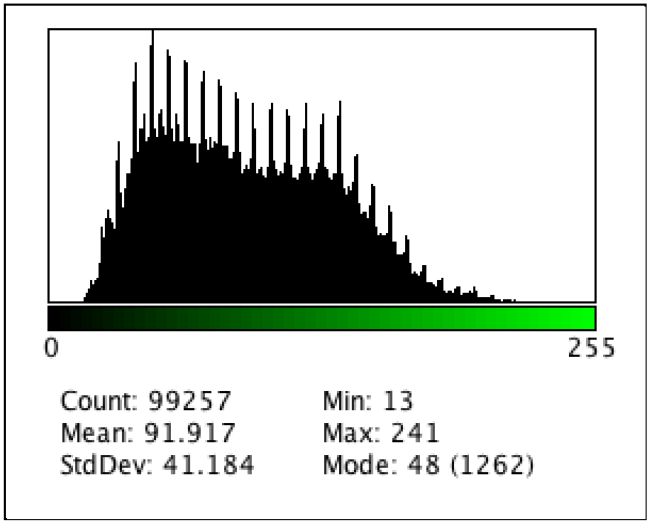

21506/+

Tumor

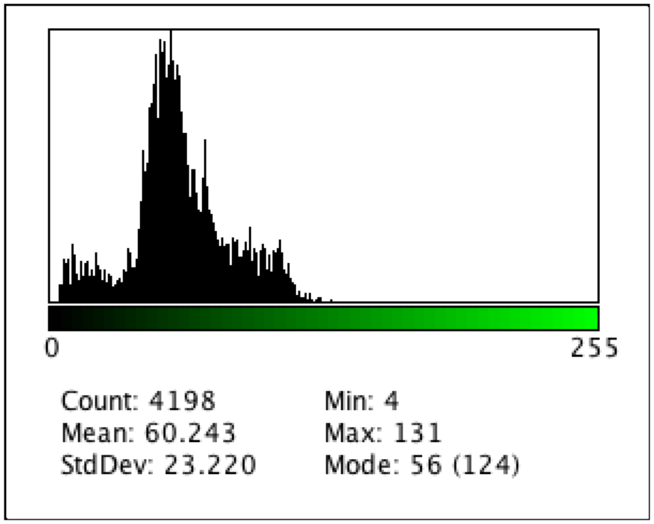

Stroma

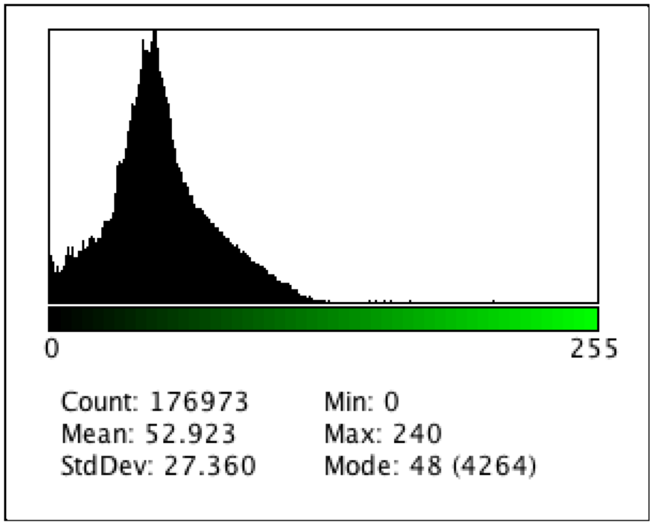

21508/+

Tumor

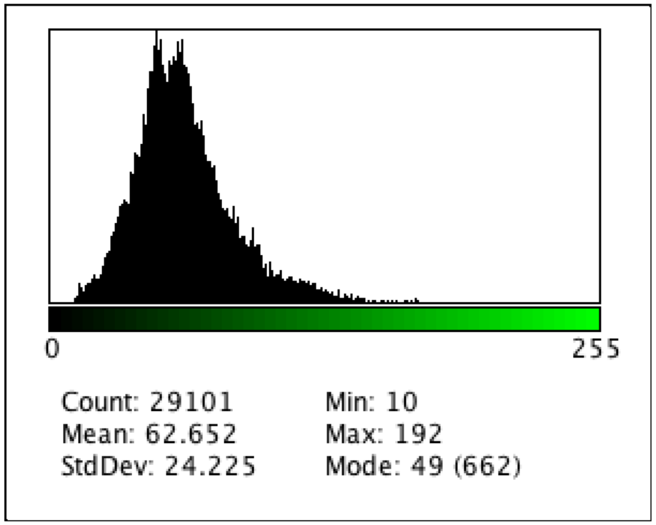

Stroma

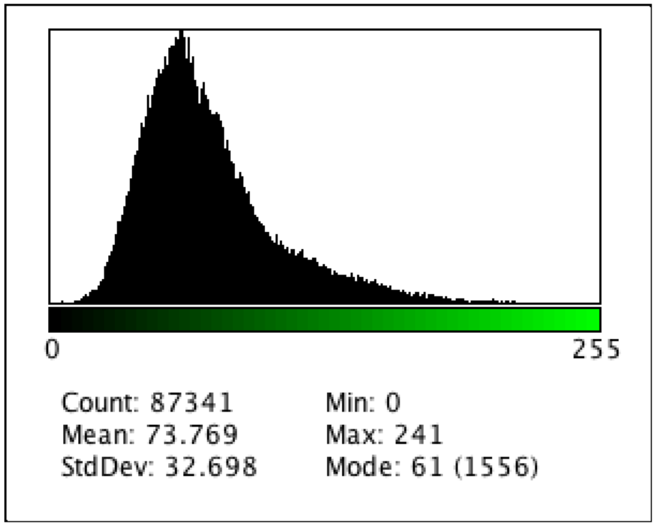

21509/+

Tumor

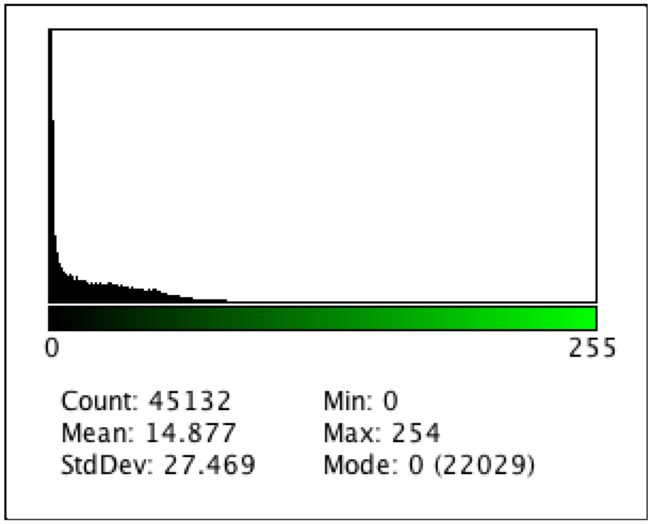

Stroma

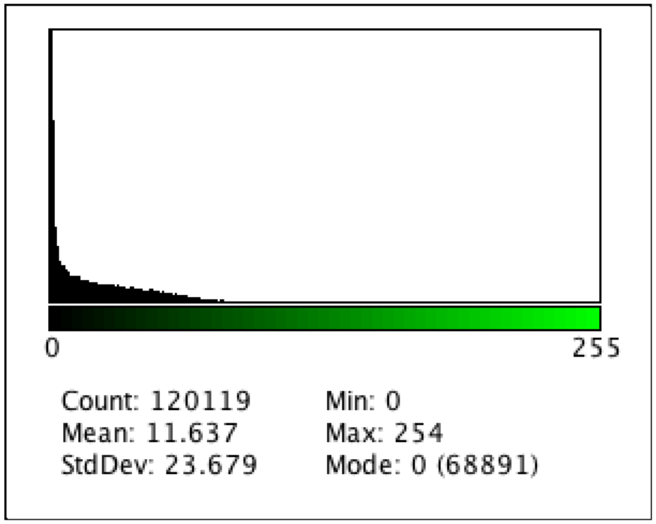

21557/+

Tumor

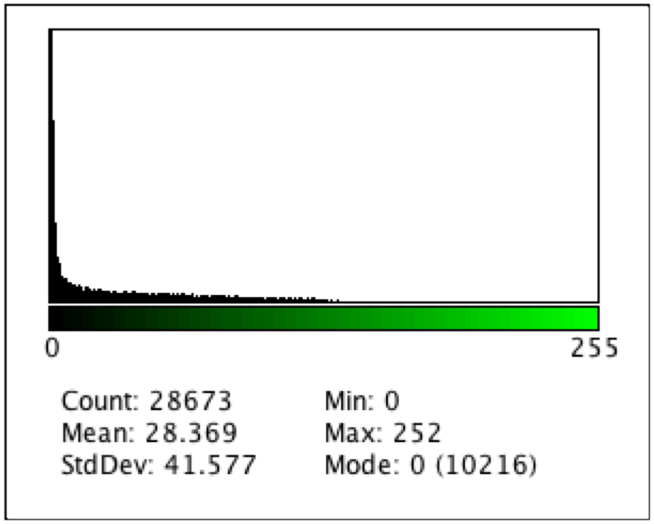

Stroma

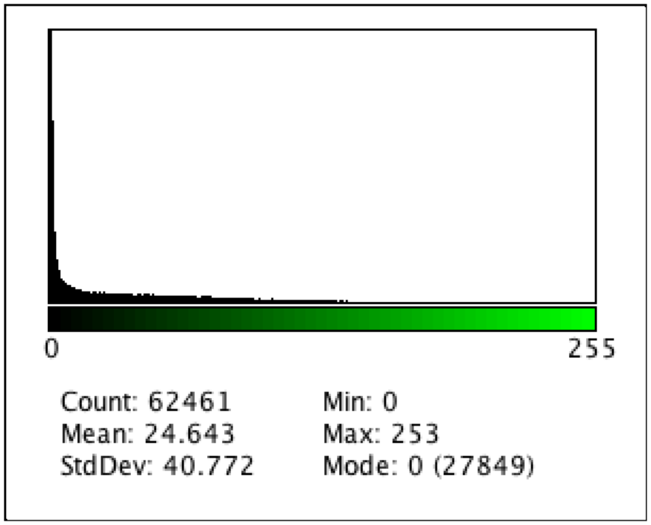

Supplement: Supplementary file 4 — 10.1186/s40169-016-0090-9 Distribution of signal intensities of tamoxifen in tumor and stroma ROIs. The mean intensities, as determined by image analysis, are presented in Table 1. [file 40169_2016_90_MOESM4_ESM.pdf]
